# *APOE*-ε4 allele[s]-associated adverse events reported from placebo arm in clinical trials for Alzheimer's disease: implications for anti-amyloid beta therapy

**DOI:** 10.3389/frdem.2023.1320329

**Published:** 2024-01-15

**Authors:** Kenichiro Sato, Yoshiki Niimi, Ryoko Ihara, Kazushi Suzuki, Atsushi Iwata, Takeshi Iwatsubo

**Affiliations:** ^1^Department of Neuropathology, Graduate School of Medicine, The University of Tokyo, Tokyo, Japan; ^2^Unit for Early and Exploratory Clinical Development, The University of Tokyo Hospital, Tokyo, Japan; ^3^Department of Neurology, Tokyo Metropolitan Geriatric Medical Center Hospital, Tokyo, Japan; ^4^Division of Neurology, Internal Medicine, National Defense Medical College, Saitama, Japan

**Keywords:** adverse event, *APOE*, Amyloid-Related Imaging Abnormalities, ARIA, Alzheimer's disease, disease-modifying therapy

## Abstract

*APOE*-ε4 allele[s] is a risk factor for Alzheimer's disease (AD) and Amyloid-Related Imaging Abnormalities (ARIA) in anti-amyloid beta therapy, and is also associated with cerebrovascular risk factors such as hyperlipidemia or atherosclerosis. During AD clinical trials, *APOE*-ε4 carriers may experience neuropsychiatric adverse events (AEs) related to these risks, complicating the differentiation of ARIA from cerebrovascular events based on symptoms. This study aimed to examine the hypothetical impact of considering the *APOE*-ε4 allele's risk for non-ARIA AEs during AD clinical trials. We used data from the Critical Path for Alzheimer's Disease (CPAD) from the placebo arm of randomized controlled trials (RCT) for AD treatment. We determined whether AEs were reported more frequently in *APOE*-ε4 carriers, quantifying with reporting odds ratio (ROR) using a mixed effect model. We also evaluated the association between ROR levels and the prior probability that an AE is symptomatic ARIA. We analyzed 6,313 patients with AD or mild cognitive impairment in 28 trials. Of the prespecified 35 neuropsychiatric or related AEs, several had a significantly high ROR: “delusion” (ROR = 4.133), “confusional state” (ROR = 1.419), “muscle spasms” (ROR = 9.849), “irritability” (ROR = 12.62), “sleep disorder” (ROR = 2.944), or “convulsion” (ROR = 13.00). However, none remained significant after adjusting for Mini-Mental State Examination scores. There is no strong evidence to suggest that specific neuropsychiatric AEs occur more frequently without drug treatment association among *APOE*-ε4 carriers. The influence of *APOE*-ε4 allele[s] on the clinicians' assessment of the likelihood of ARIA during safety monitoring in anti-amyloid beta monoclonal antibody treatment might be unchanged, thus maintaining the current level of awareness of clinicians of AEs.

## 1 Introduction

*APOE*-ε4 allele[s] is associated with a higher risk of developing Alzheimer's disease (AD) (Lumsden et al., 2020) and its earlier onset, making it a critical focus in observational studies on AD or dementia. It is also associated with an increased risk of developing Amyloid-Related Imaging Abnormalities (ARIA) in clinical trials involving anti-amyloid beta therapy, which is a disease-modifying therapy (DMT) aimed at treating or preventing AD (Sperling et al., 2011; Cummings et al., 2021; Barakos et al., 2022). Notable examples of these therapies include aducanumab (Cummings et al., 2021) and lecanemab (Cummings et al., 2023), which have recently been approved. Consequently, the *APOE* genotype is of considerable importance to clinicians and investigators during safety monitoring in clinical trials or clinical practice.

ARIA is typically classified into two main types based on imaging findings: ARIA-H (hemorrhage) and ARIA-E (edema/effusion), and they can coexist with each other. ARIA-H includes microhemorrhages and superficial siderosis, manifested as hypointense foci on T2^*^-weighted gradient-recalled echo and susceptibility-weighted (SWI) magnetic resonance imaging (MRI) scans (Roytman et al., 2023). ARIA-E represents vasogenic edema or sulcal effusion, identified by hyperintensities on fluid-attenuated inversion recovery (FLAIR) MRI scans (Roytman et al., 2023). While many ARIA findings are asymptomatic, some present with specific or non-specific symptoms such as headahe, confusion, nausea, visual disturbanes, or seizures (Cummings et al., 2023). Routine MRI scans need to be scheduled for patients who are to receive aducanumab or lecanemab treatment to detect even asymptomatic ARIA at the appropriate time.

Furthermore, the presence of the *APOE*-ε4 allele[s] has been reported to increase the risk of various disease statuses, such as hyperlipidemia (HL) (e.g., weighted mean difference +0.1~0.3 mmol/L compared to those with ε3/ε3) (Bennet et al., 2007; Khan et al., 2013), increased carotid artery intima-media thickness (e.g., by +0.3 mm compared to those with ε3) (Elosua et al., 2004), carotid and coronary atherosclerosis (Granér et al., 2008), increased white matter lesions (de Leeuw et al., 2004), or increased risk of coronary diseases (e.g., odds ratio 1.06 compared to those with ε3/ε3) (Bennet et al., 2007). These conditions are also risk factors for cerebrovascular diseases. Despite inconsistent findings in earlier studies, ischemic stroke has also been associated with the *APOE*-ε4 allele[s] (e.g., odds ratio of approximately 2 compared to those with ε3) (Kokubo et al., 2000; MacLeod et al., 2001; Abboud et al., 2008; Chen and Hu, 2016). In light of this, clinical trial participants with *APOE*-ε4 allele[s] may experience additional adverse events (AEs) related to cerebrovascular diseases (Tai et al., 2016), a risk that remains regardless of their group allocation in the trials.

This risk of cerebrovascular or other AEs due to *APOE*-ε4, which is referred to here as the “inherent risk of *APOE*-ε4,” has not been seriously recognized. However, it may pose challenges to clinical inference during safety monitoring in trials or clinical practice of DMT drugs. This is due to the wide variety of ARIA symptoms that can mimic cerebrovascular events or other neuropsychiatric symptoms and vice versa. For example, suppose that there is a participant with *APOE*-ε4 allele[s] who has recently developed symptomatic AE that is typically observable as one of the ARIA symptoms (e.g., seizure or confusion) during a randomized controlled trial (RCT); elective MRI scans may be required even if it is out-of-schedule. Meanwhile, when there is a participant with *APOE*-ε4 allele[s] who has recently developed a symptomatic AE that is not typical as one of the ARIA symptoms (e.g., fatigue or tremor), it is up to the clinicians to decide whether to perform MRI scans. Furthermore, suppoe there is a patient with *APOE*-ε4 allele[s] who has recently developed symptomatic AE that may be an ARIA symptom but is highly suggestive of acute ischemic stroke (e.g., hemiplegia and dysarthria); in this case, urgent brain imaging may be required immediately.

If such AE terms actually turned out to be highly reported in clinical trials in association with having *APOE*-ε4 allele[s] itself, regardless of its actual causality, the AE observed in participants with *APOE*-ε4 allele[s] shall be less attributed to ARIA compared to the conventional assumption in which the “inherent AE risk of *APOE*-ε4 allele[s]” is not considered or ignored. This means that clinicians' assessment of the need to perform brain imaging for ARIA detection can be influenced in the case of such AEs.

Therefore, understanding the degree of contribution of having *APOE*-ε4 allele[s] to the observed reporting of AEs, including cerebrovascular symptoms during clinical trials, might be informative for clinicians and investigators of clinical trials for AD, in a viewpoint to help distinguish some potentially confusing AEs from the symptoms due to ARIA. This could be especially true in some trials designed to concentrate participants with *APOE*-ε4 allele[s] (Vandenberghe et al., 2016). In this study, we evaluated these points using a large database of data collected from a placebo arm of RCT for the treatment of AD.

## 2 Methods

This retrospective study analyzed publicly available databases and was approved by our institutional review board. Informed consent was not obtained from participants in this study. The purpose of this study is to measure the degree of contribution of having *APOE*-ε4 allele[s] to the reporting of AEs, including cerebrovascular symptoms during clinical trials, discussing the actual necessity of considering such *APOE*-ε4-related risk of AEs in safety monitoring. For this purpose, we will utilize data from the placebo arms of earlier RCT for AD. By using data from placebo groups, we can avoid the need to consider the potential confounding effects of the adiministered active agents on the observed AEs, which could otherwise complicate the discussion.

First, we will present rationales for our purpose in the following formulations, in terms of the above consideration of the inherent *APOE*-ε4-related risk of AE involved in the safety monitoring—symptom-based vigilance of ARIA. The data used will also be described in this section.

### 2.1 Formulation (1): participants classification by virtual causality

First, consider a participant with *APOE*-ε4 allele[s] who recently developed a particular AE (referred to here as “AE_X_”), which may or may not be attributed to ARIA in the brain. Examples of AEx include headaches, nausea, and dizziness (Sperling et al., 2011; Barakos et al., 2022). These events occurred during an RCT in which participants with mild cognitive impairment (MCI) or AD, with or without *APOE*-ε4 allele[s], are randomly assigned to an active arm (e.g., receiving anti-amyloid monoclonal antibody) or a placebo arm in an arbitrary proportion. At the end of the trial, all participants were classified into two AE types (Table 1): (a) cases who had ever reported an AE_X_ during the trial and (b) cases who had never reported an AE_X_.

**Table 1:** Classification of participants and their frequency by trial arm, *APOE* status, and AE reporting.

***APOE* (w/wo ε4)**	**AE causality type**	**Active arm (**ε**4**+ **:** ε**4**− ** = 1:***k*****)		**Placebo arm (**ε**4**+ **:** ε**4**− ** = 1:***j*****)	
		**(a) Cases reported AE**_X_	**(b) Cases not reported AEx**	**(a) Cases reported AE**_X_	**(b) Cases not reported AEx**
ε4 (+)	(i) AE (excluding ARIA) due to baseline risk	*aN*_*T*_ *p*_1_	*aN*_*T*_(1−*p*_1_−*p*_2_−*p*_3_−*p*_4_−*p*_5_)	*N*_*T*_ *p*_1_	*N*_*T*_(1−*p*_1_−*p*_2_−*p*_3_)
	(ii) AE (excluding ARIA) due to ε4(+)	*aN*_*T*_ *p*_2_		*N*_*T*_ *p*_2_	
	(iii) Spontaneous ARIA	*aN*_*T*_ *p*_3_		*N*_*T*_ *p*_3_	
	(iv) ARIA due to drug	*aN*_*T*_ *p*_4_		–	
	(v) Additional ARIA due to ε4(+)	*aN*_*T*_ *p*_5_		–	
ε4 (−)	(i) AE (excluding ARIA) due to baseline risk	*akN*_*T*_ *p*_1_	*akN*_*T*_(1−*p*_1_−*p*_3_)	*jN*_*T*_ *p*_1_	*jN*_*T*_(1−*p*_1_−*p*_3_)
	(ii) AE (excluding ARIA) due to ε4(+)	–		–	
	(iii) Spontaneous ARIA	*akN*_*T*_ *p*_3_		*jN*_*T*_ *p*_3_	
	(iv) ARIA due to drug	*akN*_*T*_ *p*_4_		–	
	(v) Additional ARIA due to ε4(+)	–		–	

AEs, adverse events; ARIA, amyloid-related imaging abnormality.

Participants in the active arm and fall under the AE-type (a) are further subdivided based on a the hypothetical assumption that the causes of the AE_X_ can be categorized into one of five causality types: (i) AEs attributed to baseline risk due to age or comorbidities; (ii) additional AEs (other than ARIA) attributed to having *APOE*-ε4 allele[s]; (iii) spontaneous ARIA observed regardless of the use of DMT drugs (Antolini et al., 2021); (iv) symptomatic ARIA induced by the use of DMT; and (v) additional symptomatic ARIA as a result of interaction between the anti-amyloid monoclonal antibody and having *APOE*-ε4 allele[s] (Sperling et al., 2011; Barakos et al., 2022). Please note that participants who did not report AE_X_ during the clinical trial must be categorized as AE-type (b), even if they had unrecognized “asymptomatic” ARIA in their brains.

We suppose that the total number of trial participants in ε4 (+)-placebo arm is *N*_*T*_, the total number of trial participants in ε4 (+)-active arm is *aN*_*T*_, and the proportion of participants who are classified as causality-type (i)–(v) in AE-type (a) is *p*_1_~ *p*_5_ (0 ≤ *p*_*i*_ ≤ 1 *for i* = 1 *to* 5, 0 ≤ *p*_1_ + *p*_2_ + *p*_3_ + *p*_4_ + *p*_5_ ≤ 1). The number of remaining participants with AE-type (b) in the active arm is *aN*_*T*_(1 − *p*_1_ − *p*_2_ − *p*_3_ − *p*_4_ − *p*_5_).

The total number of trial participants in ε4(–) arms is determined to *akN*_*T*_ (*k* ≥ 0) in the active arm and *jN*_*T*_ (*j* ≥ 0) in the placebo arm. AEs observed in the placebo arms are limited to AE-types (i), (ii), or (iii) because no active drugs are administered. In contrast, the AEs observed in ε4(–) arms should be AE-types (i), (iii), or (iv), thus defining the numbers in the left cells accordingly. Under the traditional assumption, which does not account for or neglect the inherent risk of AE due to *APOE*-ε4, AE-type (ii) becomes negligible, implying *p*_2_ = 0 in Table 1. It is important to emphasize that the causality-type categorizations (i)–(v) are theoretical and are intended to explain the rationale of our study. This classification is practically impossible; thus, it is not applied to the actual data analysis.

### 2.2 Formulation (2): positive predictive value

Based on the contingency table (Table 1), our goal is to estimate how many of the presented AE_X_ are attributable to ARIA in the context of safety monitoring. Specifically, we calculate the “positive predictive value (PPV)” of AE-based vigilance for ARIA. PPV is obtained using the following formula, which varies depending on whether the *APOE*-ε4 status of each participant is known to clinicians:

- PPV in a group of which participants are known to have *APOE*-ε4 allele[s]:

$$\begin{array}{l} PPV_{1}=\frac{\left(a+1\right)N_{T}p_{3}+aN_{T}p_{4}+aN_{T}p_{5}}{aN_{T}\left(p_{1}+p_{2}+p_{3}+p_{4}+p_{5}\right)+N_{T}\left(p_{1}+p_{2}+p_{3}\right)} \end{array}$$

- PPV in a group of which participants are known not to have *APOE*-ε4 allele[s]:

$$\begin{array}{l} PPV_{2}=\frac{\left(ak+j\right)N_{T}p_{3}+akN_{T}p_{4}}{\left(ak+j\right)N_{T}p_{1}+\left(ak+j\;\right)N_{T}p_{3}+akN_{T}p_{4}} \end{array}$$

- PPV in a group in which the participants' *APOE* status is unknown to investigators or clinicians:

$$\begin{array}{l} PPV_{3}=\;\frac{Numerator\;of\;PPV_{1}+Numerator\;of\;PPV_{2}}{Denominator\;of\;PPV_{1}+Denominator\;of\;PPV_{2}}. \end{array}$$

We define *p*_2_ = *r*(*p*_1_+*p*_3_) (*r* ≥ 0) for ease of subsequent calculations, in which *r* = 0 corresponds to the conventional assumption where inherent AE risk due to *APOE*-ε4 is not considered or is ignored. By incorporating the variable “*r*,” the degree of inherent risk of AE due to *APOE*-ε4 can be captured as a ratio to the baseline risk of AE not related to *APOE*-ε4. In the above equations, *a, k, j*, and *p*_1_-*p*_5_ depend on the background population of the study participants, as well as the characteristics of the administered drugs; therefore, we regard them other than *r* (and *p*_2_) as constant. The ratio of the PPVs with respect to those at *r* = 0 (= PPV_r = 0_) are as follows:

-
$PPV\;Ratio_{1}=\;\frac{PPV_{1}}{PPV_{r=0}}$

$=\frac{\left(1+a\right)\;\left(p_{1}+\;p_{3}\right)\;+a\left(p_{4}+\;p_{5}\right)}{\left(1+a\right)\;\left(1+r\right)\;\left(p_{1}+\;p_{3}\right)\;+a\left(p_{4}+\;p_{5}\;\right)}$

-
$PPV\;Ratio_{2}=\frac{PPV_{2}}{PPV_{r=0}}=1$

-
$PPV\;Ratio_{3}=\;\frac{PPV_{3}}{PPV_{r=0}}$

$=\frac{\left(1+a+ak+j\right)\;\left(p_{1}+\;p_{3}\right)\;+a\left(1+k\right)p_{4}+\;ap_{5}}{\left\{1+a+ak+j+a\;\left(1+r\right)\right\}\;\left(p_{1}+\;p_{3}\right)\;+a\left(1+k\right)\;p_{4}+\;ap_{5}}.$

All PPV ratios are equal to 1 when *r* = 0. Because of the underlined terms, both PPV Ratio_1_ and Ratio_3_ monotonically decrease as *r* increases (*r* ≥ 0). This means that, in a trial group consisting of participants with *APOE*-ε4(+) or in a group where the *APOE*-ε4 status of participants is unknown, the PPV for predicting the likelihood of ARIA in participants reporting the AE_X_ will invariably decrease to an uncertain extent when *r* > 0, compared to *r* = 0.

### 2.3 Formulation (3): reporting odds ratio

We turn our attention to Table 2, which is derived from the placebo arms presented in Table 1. The items in Table 2 are condensed into a 2-by-2 contingency table. This is done by grouping all participants in the placebo arm based on whether they reported AE_X_ (either with or without) and on their status of *APOE* (with or without ε4 allele[s]). Subsequently, the association of AE development with the *APOE*-ε4 status can be quantified as the reporting odds ratio (ROR). This metric is commonly used in pharmacovigilance studies (Sato et al., 2020) and is calculated using the following formula with the values of *N*_A_-*N*_D_ in Table 2:

-
$ROR=\frac{N_{A}/N_{B}}{N_{C}/N_{D}}.$

**Table 2:** Contingency table within the placebo arm.

		**Placebo arm (**ε**4**+**:**ε**4–** = 1:***j*****)**	
		**(a) Cases reported AE_X_**	**(b) Cases not reported AEx**
*APOE* (w/wo ε4)	ε4 (+)	*N*_*A*_ = *N*_*T*_(*p*_1_+*p*_2_+*p*_3_)	*N*_*B*_ = *N*_*T*_(1−*p*_1_−*p*_2_−*p*_3_)
ε4 (-)	*N*_*C*_ = *jN*_*T*_(*p*_1_+*p*_3_)	*N*_*D*_ = *jN*_*T*_(1−(*p*_1_+*p*_3_))

AEs, adverse events.

A significantly high ROR means that AE is reported more frequently among individuals with *APOE*-ε4 allele[s] compared to those without it, regardless of a direct causal relationship. Because the ROR reflects only reported AEs, it is susceptible to reporting bias. Consequently, the ROR differs from the OR typically measured in observational studies. The ROR was calculated using the values in Table 2 as follows:

-
$ROR=\frac{N_{T}\left(p_{1}+p_{2}+p_{3}\right)jN_{T}\left\{1-\left(p_{1}+p_{3}\right)\right\}}{\;\left[N_{T}\left\{1-p_{1}-p_{2}-p_{3}\right\}jN_{T}\left(p_{1}+p_{3}\right)\;\right]}$

Because *p*_2_ = *r*(*p*_1_+*p*_3_) and therefore *p*_1_+_*p*_2_+*p*3_ = (1+*r*)(*p*_1_+*p*_3_ ),

-
$ROR\;=\frac{\left(1+r\right)\;\left\{1-\left(p_{1}+p_{3}\right)\right\}\;}{\left(1+r\right)\;\left\{1-\left(p_{1}+p_{3}\right)\right\}-r}$

We can ascertain that ROR = 1 when *r* = 0.

Because $\frac{d}{dr}\;ROR=\frac{1-\left(p_{1}+p_{3}\right)}{\;{\left\{1-\;\left(1+r\right)\;\left(p_{1}+p_{3}\right)\right\}}^{2}}>0$ (*r* > 0), the ROR exhibits a monotonic increase along with the increasing *r* (*r* > 0). If we assume that (*p*_1_+*p*_3_) is sufficiently small that it can be approximated as 1−(*p*_1_+*p*_3_) ≅ 1, then above ROR can be further simplified as follows:

- *ROR* ≅ 1+*r*.

### 2.4 Relationship between PPV and ROR

Because the *PPV ratio* and *r* mentioned above cannot be directly obtained from actual data, we relied on another reference point: ROR = 1. When analyzing a specific AE with ROR > 1, it is inferred that *r* > 0 for that AE. Concurrently, the PPV ratio of the corresponding AE should be lower than that at *r* = 0. Specifically, discovering an AE with an ROR significantly >1 through statistical analysis of the actual data indicates a non-negligible *r* value (i.e., greater than zero). Consequently, the AE-based PPV should be lower than it would be under the conventional assumption where the inherent risk of *APOE*-ε4 is not considered or is ignored, regardless of the *p*_1_ or *p*_3_ values. It should be noted that, in this approach, determining the actual degree of PPV decline remains elusive as it depends on the values of *p*_2_, *p*_4_, and *p*_5_. Despite this limitation, this methodology can offer valuable insights for clinicians and help to identify AEs that require less vigilance when monitoring ARIA.

### 2.5 Database used

We used data from the Critical Path for Alzheimer's Disease (CPAD) (Ito et al., 2013; Neville et al., 2015; Arnerić et al., 2018). This dataset comprises thousands of participants with AD or MCI from the placebo arms of numerous RCTs aimed at AD treatment. The names of the administered drugs or trials have not been disclosed. Most participants were diagnosed with AD at baseline, while a small proportion had MCI (c.f., *PRIMARY DIAGNOSIS* in the data file named “MH”). The diagnostic criteria for AD or MCI are uncertain and may vary across studies, and we assumed that many of the enrolled participants were clinically diagnosed with AD as defined by the NINCDS-ADRDA criteria (McKhann et al., 1984). We also retrieved baseline data on the use of symptomatic anti-dementia drugs prior to the beginning of trials, including donepezil, galantamine, rivastigmine, and memantine. The severity of AD or cognitive scores, such as Mini-Mental State Examination (MMSE) at baseline, was provided only in a subset of the included studies (c.f., the variable *QSSTRESC* in the data file named “QS”).

AEs that appeared in the early phase would hardly be ARIA, as most of the development of ARIA was observed in aducanumab or lecanemab trials 8–9 weeks after the beginning of drug administration (Barakos et al., 2022; Honig et al., 2023). Consequently, we excluded AEs that emerged 4 weeks after the start of the study from the analysis. We included AEs of any severity, although ARIA symptoms observed in the aducanumab or lecanemab trials were reportedly mostly mild (Barakos et al., 2022; Honig et al., 2023).

### 2.6 Data analyses

We want to obtain the ROR of having *APOE*-ε4 to the reporting/development of AE (in binary: with/without) while adjusting for age at baseline, sex, and other variables. Among the reported AEs, we arbitrarily selected 35 that may be associated with neuropsychiatric or cerebrovascular symptoms. The 35 AEs are listed in Supplementary Table 1. Among them, we analyzed AEs, the frequency of which was ≥10 in the examined data.

We used a mixed effects model (Bates et al., 2015) by appointing the individual study ID as a random intercept because the CPAD database used is the aggregated data of different RCTs with which the inclusion criteria, background population, length of period, or administered drugs differ one by one, and there should be some heterogeneity in the AE reports of each RCT study. The equations for the generalized linear mixed model are as follows:

- Model (1): log(*Odds*) = β_0_ + *Age*·β_1_ + *Sex*·β_2_ + *APOE*(*e*4)·β_3_ + *Drug*·β_4_ + *Diagnosis*·β_5_ + *Interaction*(*age* × *APOE*)·β_6_ + *Study*·γ_0_
- Model (2): log(*Odds*) = β_0_ + *Age*·β_1_ + *Sex*·β_2_ + *APOE*(*e*4)·β_3_ + *Drug*·β_4_ + *Diagnosis*·β_5_ + *Interaction*(*age* × *APOE*)·β_6_ + *MMSE*·β_7_ + *Study*·γ_0_

where β_0_ is the fixed intercept, *Age* is the age of participants at index starting of the trial, *Sex* is a binary variable (male or female), *APOE*(ε4) is a binary variable whether each participant has *APOE*-ε4 allele[s], *Drug* refers to a binary variable showing a medical history of taking any drugs related to dementia treatment (i.e., donepezil, galantamine, rivastigmine, or memantine), *Diagnosis* refers to binary variable showing diagnosis of dementia of each participant, *MMSE* refers to baseline MMSE total score of each participant, and γ_0_ denotes random intercept by each study (Sato et al., 2021). We included the interaction term between age and *APOE* genotype to separately analyze *APOE*-related amyloid burden, which should be exacerbated with age. In Model (2), we included MMSE scores, as different cognitive statuses may lead to different symptoms, represented as AEs. As underlined in the formulas, β_3_ is the coefficient we want to obtain. When the lower 95% confidence interval (CI) of the exp(β_3_) is higher than 1, the *OR*_*AE*_*X*__ is considered significantly high.

## 3 Results

### 3.1 Data summary

A total of 28 clinical trial data were included in the eligible CPAD data, with 6,313 participants (Supplementary Table 2). Approximately 85% of the participants had AD at baseline and the remaining had MCI. Their median age was 75.0 years old (95% CI, 69.0–80.0), and 56.2% of them were female. Among all participants, 24.4% had one or two *APOE*-ε4 allele[s], and those with *APOE*-ε4 allele[s] were slightly older than those without them (median 75.0 years old vs. 74.0 years old, *p* = 0.001, Wilcoxon rank sum test), while no association was observed between the frequency of sex and *APOE*-ε4 allele[s] (*p* = 0.097, chi-square test).

In addition, we summarized the basic characteristics of the subgroups in models (1) and (2) (Supplementary Table 3). In summary, the cases included in model (2) were more prevalent in having *APOE*-e4 carriers (24.4% vs. 47.7%) and had a primary diagnosis of AD (86.1% vs. 100%) compared to those in model (1). The MMSE scores for cases in model (2) had a median of 20 (IQR: 19 ~ 22).

### 3.2 Identified AEs

Among the 35 prespecified AEs examined (Supplementary Table 1), 29 AEs with a frequency of ≥10 within the examined data were examined with Model (1), and only 15 AEs with a frequency of ≥10 within the examined data were examined with Model (2). Please note that Model (2) requires the MMSE score so that participants whose MMSE was not recorded were excluded from the analysis. Some AEs such as “delusion” (ROR = 4.133), “confusional state” (ROR = 1.419), “muscle spasms” (ROR = 9.849), “irritability” (ROR = 12.62), “sleep disorder” (ROR = 2.944), and “convulsion” (ROR = 13.00) were identified as those with significantly high ROR (lower 95% >1) by Model (1). However, none of them were confirmed to be significant when examined using Model (2) (Table 3).

**Table 3:** Results of the analysis of two different models.

**AE term**	**Model (1) (*n* = 6,313) Adjusted OR (95% CI)**	**Model (2) (*n* = 1,303) Adjusted OR (95% CI)**
Fall	1.088 (0.756–1.568)	1.334 (0.581–3.066)
Dizziness	1.07 (0.656–1.744)	0.963 (0.406–2.28)
Nausea	1.187 (0.7–2.013)	1.215 (0.457–3.233)
Agitation	1.177 (0.683–2.027)	2.262 (0.642–7.975)
Headache	0.758 (0.438–1.311)	0.821 (0.323–2.086)
Depression	0.813 (0.448–1.475)	1.075 (0.416–2.775)
Vomiting	0.918 (0.48–1.757)	1.08 (0.3–3.883)
Insomnia	1.05 (0.561–1.966)	0.919 (0.269–3.133)
Depressed mood	1.043 (0.517–2.106)	NA
Restlessness	1.512 (0.679–3.368)	NA
Anxiety	1.087 (0.555–2.132)	0.513 (0.179–1.471)
Somnolence	0.498 (0.252–0.982)	NA
Decreased appetite	1.635 (0.701–3.813)	1.079 (0.388–2.999)
Delusion	4.133 (1.301–13.13)^*^	2.106 (0.35–12.671)
Fatigue	1.583 (0.68–3.687)	1.478 (0.501–4.363)
Aggression	1.616 (0.689–3.786)	2.173 (0.438–10.77)
Confusional state	1.419 (1.415–1.424)^*^	1.173 (0.286–4.818)
Hallucination	1.837 (0.643–5.248)	NA
Muscle spasms	9.849 (1.879–51.611)^*^	NA
Tremor	3.581 (0.706–18.159)	NA
Delirium	2.84 (0.789–10.229)	NA
Gait disturbance	1.114 (0.281–4.419)	NA
Irritability	12.62 (2.313–68.861)^*^	NA
Sleep disorder	2.944 (1.082–8.012)^*^	NA
Convulsion	12.995 (1.652–102.197)^*^	NA
Vertigo	0.572 (0.212–1.549)	0.429 (0.098–1.872)
Depressive symptom	1.628 (0.399–6.638)	NA
Cerebrovascular accident	0.606 (0.164–2.233)	NA
Balance disorder	NA	NA
Disorientation	NA	NA
Transient ischemic attack	1.037 (0.206–5.214)	NA
Nightmare	NA	NA
Vision blurred	NA	NA
Abnormal behavior	NA	NA
Cognitive disorder	NA	NA

AEs with a frequency of ≤ 10 within the data examined are not examined (as shown with “NA” in the table). AEs whose OR is significantly high ORs (>1) are indicated by asterisks (^*^).

AEs, adverse events; OR, odds ratio; CI, confidence interval.

## 4 Discussion

In this study, we presented a hypothetical formulation to examine how the “inherent *APOE*-ε4 related risk of AE” may potentially influence the symptom-based vigilance of ARIA during safety monitoring. We then assessed the degree of impact using actual data analysis. As a result, regardless of their causal relationship, we found that certain neuropsychiatric AEs, namely “delusion,” “confusional state,” “muscle spasms,” “irritability,” “sleep disorder,” or “convulsion,” may be reported more frequently by individuals with the *APOE*-ε4 allele[s] among placebo arm during RCTs [Table 3, Model (1)]. However, these findings were not consistent when adjusted for baseline MMSE scores [Table 3, Model (2)].

Although the cases included in model (2) were more prevalent in having *APOE*-e4 carriers and had a primary diagnosis of AD compared to those in model (1), the differences in the characteristics of the participants included in the models (1) and (2) do not undermine our conclusion; instead, they contribute to enhancing its robustness. Furthermore, although the cases included in model (2) (i.e., MMSE median 20) may be slightly more severe than those for whom currently available DMT drug such as lecanemab is typically indicated (e.g., MMSE 22–30) (Cummings et al., 2023), we consider this justifiable since such a level of change in MMSE might be observed over the course of the disease, even in those who began treatment with lecanemab.

Collectively, these results suggest that there is currently no solid statistical evidence indicating that some neuropsychiatric AEs are more likely to be reported by individuals with the *APOE*-ε4 allele[s] during RCTs solely in association with the *APOE*-ε4 allele[s] but not with the development of ARIA. According to our formulation, this means that there is no reliable AE whose *r* > 0, then it is implied that AEs due to *APOE*-ε4 by itself might not influence investigators to consider the probability of being ARIA in safety monitoring during clinical trials with anti-amyloid beta monoclonal antibodies (i.e., PPV_ratio_ = 1). The level of alertness required for clinicians to these AEs might be unchanged even when considering the inherent *APOE*-ε4-related risk of AE after all.

The unique contribution of our study lies in its focus on the AE risk associated solely with *APOE*-ε4, a factor that has often been overlooked. In clinical trials involving anti-amyloid monoclonal antibody medications, the predominant concern has been the interactive risk of ARIA with *APOE*-ε4: the development of ARIA is a significant safety concern when monitoring anti-amyloid monoclonal antibodies (Sperling et al., 2011; Cummings et al., 2021, 2023; Barakos et al., 2022). Although asymptomatic ARIA occurs more frequently than symptomatic ARIA, many cases of symptomatic ARIA have been reported to be mild (Barakos et al., 2022). Regular MRI evaluations are typically set on predefined schedules (Cummings et al., 2021, 2023), but additional MRI scans may be warranted, especially when the observed AEs resemble ARIA symptoms during trials. PPV evaluated in this study served as a metric to measure the likelihood of ARIA in these clinical scenarios. In this study, we confirmed that clinicians' judgment on the need for brain imaging to detect ARIA remains unchanged.

The sensitivity and specificity to identify ARIA among individuals with certain AEs should be considered instead of PPV, as in this study. However, because not all ARIAs are detected/reported in clinical trials due to the varying frequency of MRI assessments, it is challenging to compare the development of AEs between participants with and without ARIA. Furthermore, asymptomatic ARIA cannot be captured using our current AE-based approach. Therefore, we chose not to use sensitivity or specificity as metrics to measure the utility of our approach. In future research, it will be essential to obtain sensitivity, specificity, or other classification metrics for individual AEs when predicting symptomatic ARIA, especially if evidence is available on the timing of ARIA development for each anti-amyloid agent or consistent MRI scheduling protocols.

There are some limitations to this study. In particular, we at first wanted to incorporate variables associated with the severity of AD pathology because such variables might also be associated with the development of ARIA or related AEs. However, they were not available in the used data. Instead, we referred to MMSE, a cognitive score, as a surrogate variable which has correspondence with the clinical severity of AD. Incorporating MMSE scores as a result greatly reduced the number of cases available for analysis [*n* = 6,313 in Model (1) and *n* = 1,303 in Model (2)]. Participants were clinically diagnosed with AD or MCI; however, they were not always diagnosed based on specific AD biomarkers. The prevalence or degree of AD pathology shall be higher in a subgroup with *APOE*-ε4 allele[s] than in those without, which means that the ROR of AEs measured in this study may partially reflect the effect of developing AE by mixed-in non-AD pathology or by the degree of amyloid or tau burden (Sato et al., 2019). We attempted to ameliorate this potential confounding by including an interaction term between age and *APOE* genotype, although we could not consider other factors, such as hypertension or diabetes mellitus, that can exacerbate cerebrovascular damage due to *APOE*-ε4 (Tai et al., 2016) in the models because of the lack of these variables.

In future studies, replicating the current results could be beneficial using datasets from the placebo arms of RCTs for various anti-amyloid drugs similar to those of the CPAD, such as the YODA Project (https://yoda.yale.edu), which includes the placebo arms from two RCTs of bapineuzumab, one of the anti-amyloid drugs for AD. In future, we would like to conduct a comprehensive validation study, including the placebo arms of RCTs for several kinds of anti-amyloid drugs, as soon as more RCT data becomes publicly available.

Furthermore, although we examined the association between having one or more *APOE*-ε4 alleles and the development of AEs, the association between possessing two *APOE*-ε4 alleles and the subsequent development of AEs also requires evaluation. This is because the frequency of ARIA increases significantly in individuals homozygous for the *APOE*-ε4 allele when administered anti-amyloid drugs, which results in a matter of critical decision-making regarding the initiation of treatment with DMTs for those carrying the *APOE*-ε4 homozygous genotype. Due to the limitations of the formulations we used, we could not assess this; therefore, we may need to develop new formulations that can take into account the number of *APOE*-ε4 alleles and the development of AEs in future studies.

In conclusion, we presented a formulation to determine how the inherent *APOE*-ε4 related risk of AE might potentially influence the symptom-based vigilance of ARIA during safety monitoring. As a result, there is no strong evidence suggesting that specific neuropsychiatric AEs are more common in *APOE*-ε4 carriers of the placebo arm during RCTs. The *APOE*-ε4 allele's influence on the likelihood of ARIA during safety monitoring in anti-amyloid beta monoclonal antibody trials could be negligible, after all, maintaining the current level of clinician alertness.

## Data availability statement

Publicly available datasets were analyzed in this study. This data can be found at: https://c-path.org/programs/cpad/.

## Ethics statement

Ethical review and approval was not required for the study on human participants in accordance with the local legislation and institutional requirements. Written informed consent was not required to participate in this study in accordance with the local legislation and institutional requirements.

## Author contributions

KSa: Conceptualization, Data curation, Formal analysis, Funding acquisition, Methodology, Visualization, Writing – original draft. YN: Writing – review & editing. RI: Writing – review & editing. KSu: Writing – review & editing. AI: Writing – review & editing. TI: Supervision, Writing – review & editing.
