# Supplementary material for: APOE-ε4 allele[s]-associated adverse events reported from placebo arm in clinical trials for Alzheimer's disease: implications for anti-amyloid beta therapy
Source: Front Dement. 2024 Jan 15;2:1320329. doi: 10.3389/frdem.2023.1320329 (PMC11285649; doi:10.3389/frdem.2023.1320329)
Supplement: Supplementary file 1 [file Table_1.docx]

Supplementary Material

**Supplementary Table 1**. The 35 AEs examined and its frequency in the database.

| AE term | N with AE in *APOE*-ε4 (-) patients | N with AE in *APOE*-ε4 (+) patients |
| --- | --- | --- |
| Fall | 105 | 91 |
| Dizziness | 85 | 44 |
| Nausea | 82 | 39 |
| Agitation | 36 | 34 |
| Headache | 89 | 27 |
| Depression | 73 | 25 |
| Vomiting | 40 | 25 |
| Insomnia | 56 | 23 |
| Depressed mood | 20 | 23 |
| Restlessness | 15 | 22 |
| Anxiety | 47 | 21 |
| Somnolence | 37 | 19 |
| Decreased appetite | 13 | 17 |
| Delusion | 9 | 17 |
| Fatigue | 36 | 16 |
| Aggression | 13 | 16 |
| Confusional state | 14 | 12 |
| Hallucination | 7 | 12 |
| Muscle spasms | 5 | 11 |
| Tremor | 12 | 9 |
| Delirium | 6 | 9 |
| Gait disturbance | 8 | 8 |
| Irritability | 3 | 8 |
| Sleep disorder | 9 | 8 |
| Convulsion | 2 | 8 |
| Vertigo | 30 | 7 |
| Depressive symptom | 5 | 6 |
| Cerebrovascular accident | 9 | 6 |
| Balance disorder | 2 | 5 |
| Disorientation | 2 | 5 |
| Transient ischemic attack | 13 | 4 |
| Nightmare | 3 | 4 |
| Vision blurred | 5 | 3 |
| Abnormal behavior | 4 | 3 |
| Cognitive disorder | 5 | 3 |

Only AEs with frequency of ≥10 are examined.

AEs, adverse events.
