# Supplementary material for: APOE-ε4 allele[s]-associated adverse events reported from placebo arm in clinical trials for Alzheimer's disease: implications for anti-amyloid beta therapy
Source: Front Dement. 2024 Jan 15;2:1320329. doi: 10.3389/frdem.2023.1320329 (PMC11285649; doi:10.3389/frdem.2023.1320329)
Supplement: Supplementary file 2 [file Table_2.docx]

Supplementary Material

**Supplementary Table 2.** Patient characteristics.

|  | *APOE*-ε4 (-) | *APOE*-ε4 (+) |
| --- | --- | --- |
| (Total: n = 6,313) | 4774 (75.6%) | 1539 (24.3%) |
| Age (y/o) | Median 75 (IQR: 68−81) | Median 74 (IQR: 69−79) |
| Sex (female) | 2656 (55.6%) | 894 (58.1%) |
| Diagnosis (AD [or MCI]) | 4013 (84.1%) | 1420 (92.3%) |
| Medication for dementia  (with [or without]) | 2305 (48.3%) | 884 (57.4%) |

AD, Alzheimer’s disease; IQR, interquartile range; MCI, mild cognitive impairment.

Most participants were diagnosed with AD at baseline, while a small proportion had MCI (c.f., *PRIMARY DIAGNOSIS* in the data file named “MH”). The diagnostic criteria for AD or MCI are uncertain and may vary across studies. We also retlieved baseline data on the use of symptomatic anti-dementia drugs, including donepezil, galantamine, rivastigmine, and memantine.
