# Supplementary material for: APOE-ε4 allele[s]-associated adverse events reported from placebo arm in clinical trials for Alzheimer's disease: implications for anti-amyloid beta therapy
Source: Front Dement. 2024 Jan 15;2:1320329. doi: 10.3389/frdem.2023.1320329 (PMC11285649; doi:10.3389/frdem.2023.1320329)
Supplement: Supplementary file 3 [file Table_3.docx]

Supplementary Material

**Supplementary Table 3.** Patient characteristics in Models (1) and (2) and their difference.

|  | Model (1) | Model (2) | *P-value* |
| --- | --- | --- | --- |
| Total N | 6,313 | 1,303 |  |
| Age (y/o) | Median 75 (IQR: 69−80) | Median 75 (IQR: 69−80) | *0.884* |
| Sex (female) | 3550 (56.2%) | 745 (57.2%) | *0.553* |
| *APOE*-ε4 (+) | 1539 (24.4%) | 621 (47.7%) | *<0.001* |
| Diagnosis (AD [or MCI]) | 5433 (86.1%) | 1303 (100%) | *<0.001* |
| Medication for dementia  (with [or without]) | 3189 (50.5%) | 1197 (91.9%) | *<0.001* |
| MMSE total score | N/A | 20 (IQR: 16 ~ 22) | *-* |

AD, Alzheimer’s disease; IQR, interquartile range; MCI, mild cognitive impairment; MMSE, Mini-Mental State Examination.

Most participants were diagnosed with AD at baseline, while a small proportion had MCI (c.f., *PRIMARY DIAGNOSIS* in the data file named “MH”). The diagnostic criteria for AD or MCI are uncertain and may vary across studies. We also retlieved baseline data on the use of symptomatic anti-dementia drugs, including donepezil, galantamine, rivastigmine, and memantine. The Mini-Mental State Examination (MMSE) score were provided only in a subset of the included studies (c.f., the variable *QSSTRESC* in the data file named “QS”).
